# Approach for Text Classification Based on the Similarity Measurement between Normal Cloud Models

**DOI:** 10.1155/2014/784392

**Published:** 2014-02-23

**Authors:** Jin Dai, Xin Liu

**Affiliations:** College of Software Engineering, Chongqing University of Posts and Telecommunications, Chongqing 400065, China

## Abstract

The similarity between objects is the core research area of data mining. In order to reduce the interference of the uncertainty of nature language, a similarity measurement between normal cloud models is adopted to text classification research. On this basis, a novel text classifier based on cloud concept jumping up (CCJU-TC) is proposed. It can efficiently accomplish conversion between qualitative concept and quantitative data. Through the conversion from text set to text information table based on VSM model, the text qualitative concept, which is extraction from the same category, is jumping up as a whole category concept. According to the cloud similarity between the test text and each category concept, the test text is assigned to the most similar category. By the comparison among different text classifiers in different feature selection set, it fully proves that not only does CCJU-TC have a strong ability to adapt to the different text features, but also the classification performance is also better than the traditional classifiers.

## 1. Introduction

Text classification is key technology in text mining, which denotes the task of assigning raw text documents to one or more predefined categories. This is a direct concept from machine learning, which implies the declaration of a set of labeled categories as a way to represent the documents and a statistical classifier trained with a labeled training set. Classification is the process in which objects are initially recognized, differentiated, and understood and implies that objects are grouped into categories, usually for some specific purposes. Classification is fundamental in prediction, inference, and decision making. However, there are a variety of ways to approach classification task [1].

In recent years, with the continued development and innovation of information technology and natural language processing (NLP) fields, it has laid a solid theory and practice basis for text classification. An increasing number of supervised classification approaches have been developed for various types of classification tasks, such as decision trees (DT) [2, 3], neural networks (NN) [4, 5], naive Bayes (NB) [6–8], support vector machines (SVM) [9–12], and *k* nearest neighbor(*k*NN) [13–15]. These classifiers have their own characteristics, but in the perspective of comprehensive performance, SVM, *k*NN, and NB methods are more excellent [16].

SVM can handle high-dimensional sparse text and is not sensitive to the characteristics of relevance. But its classification speed is too slow and lacks the method for multi-class classification. In addition, the choice of kernel function and parameters still depended on experience [9]. The *k*NN method has the advantage of simple and stable performance and especially has good anti-interference to the noise data. But, it requires a large number of samples to get threshold. At the same time, how to choose *k* value has a direct impact on the classification performance [14]. NB is simple, efficient, and stable. It requires few parameters and is not sensitive to the missing data. However, NB assumes that the attributes are mutually independent, but in practice the assumption is often not established. Especially, if the amount of attributes is larger or the correlation is great between attributes, its efficiency is low [17].

Cloud model [18–20] is an uncertainty conversion model between the quality concepts and its quantity value expressed by the natural language value. The character of cloud can be expressed by expected value (Ex), entropy (En), and hyper entropy (He) [18]. Ex is the center value of concept in the theory field, which represents the value of the qualitative concept. En is the measuring of the fuzziness of qualitative concept, which reflects the numerical range that can be accepted by this concept in the theory field. He embodies the uncertainty of the qualitative concept. The bigger the entropy is, the bigger the numerical range can be accepted by the concept and the fuzzier the concepts are.

Given three digital features Ex, En, and He, the vector *C*(Ex, En, He) is called cloud vector, which represents a qualitative concept.

With the efficient conversion function between qualitative and quantitative data, the concept extraction method of cloud model is applied to text classification. Through the conversion from text collection to text information table based on VSM model, the text qualitative concept, which is extraction from the same category, is jumping up as a whole concept. On the basis of the cloud similarity between the test text and each category, the test text is assigned to the most similar category. By the comparison among different text classifiers based on different feature selection methods, it has not only a strong ability to adapt to the different text features, but also better classification performance than the traditional classifiers.

## 2. Similarity Measurement between Normal Cloud Models

In cloud model theory, the cloud vector $\vec{C}=\{\text{Ex},\text{En},\text{He}\}$ is used to descript the qualitative concept. When making comparison of the similarity between vectors, Euclidean Distance or Cosine Angle is widely used. However, the physical meaning and the importance of each dimensionality of the cloud vector are completely different. Therefore, a novel similarity measurement between cloud vectors is required. Taking into account the normal cloud model with most universal, we propose the similarity degree between normal clouds.

**Figure 1:**
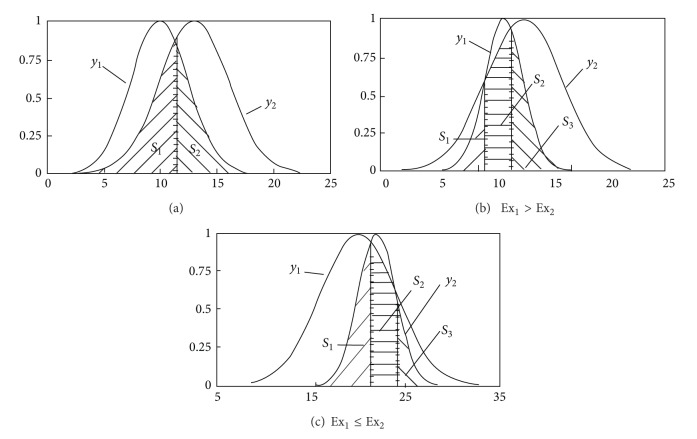
Similar situation between normal clouds.

Normal random distribution is the core of this similarity degree, which is obtained by calculating the area of two intersecting normal cloud vectors. The intersecting stations of two normal clouds are shown in Figure 1 (the shadow area presents each other's similarity).

Given ${\vec{C}}_{1}=\left\{\text{E}{\text{x}}_{1},\text{E}{\text{n}}_{1},\text{H}{\text{e}}_{1}\right\}$, ${\vec{C}}_{2}=\left\{\text{E}{\text{x}}_{2},\text{E}{\text{n}}_{2},\text{H}{\text{e}}_{2}\right\}$,  *y*_1_(*x*) and *y*_2_(*x*) are,respectively, the distribution function of ${\vec{C}}_{1}$ and ${\vec{C}}_{2}$, where *x*_0_ is the intersection of the two curves *y*_1_ and *y*_2_. Then, the intersecting area of ${\vec{C}}_{1}$ and ${\vec{C}}_{2}$ is

(1) S=∫−∞x0y2(x)dx+∫x0∞y1(x)dx=∫−∞z2ϕ(z)dz+∫z1∞ϕ(z)dz,

where *z*_1_ = (*x*_0_ − Ex_1_)/En_1_, *z*_2_ = (*x*_0_ − Ex_2_)/En_2_, *ϕ*(*z*) is standard normal distribution. If *x*_0_ is known, *z*_1_ and *z*_2_ are then obtained. Enquiring the table of standard normal distribution, the intersecting area *S* can be calculated.

On the basis of the intersection between normal cloud vectors, |*z*_1_| = |*z*_2_|, then obtains that

(2) $$\begin{array}{c} x_{0}^{\left(1\right)}=\frac{\text{E}{\text{x}}_{2}\text{E}{\text{n}}_{1}-\text{E}{\text{x}}_{1}\text{E}{\text{n}}_{2}}{\text{E}{\text{n}}_{1}-\text{E}{\text{n}}_{2}},\\ x_{0}^{\left(2\right)}=\frac{\text{E}{\text{x}}_{1}\text{E}{\text{n}}_{2}+\text{E}{\text{x}}_{2}\text{E}{\text{n}}_{1}}{\text{E}{\text{n}}_{1}+\text{E}{\text{n}}_{2}}. \end{array}$$

In light of “3En” principle of normal distribution, 99.74% of values are in [Ex − 3En, Ex + 3En]. So, when calculating the similarity of two normal distributions, we only consider the distribution of variables in the interval. It would be well as if set Ex_1_ ≤ Ex_2_; then, there are the three following situations about the distribution of *x*_0_^(1)^ and *x*_0_^(2)^.

(1) If *x*_0_^(1)^, *x*_0_^(1)^ ∉ [Ex_2_ − 3En_2_, Ex_1_ + 3En_1_], indicating that the values distribution of intersections can be neglected, so *S* = 0.

(2) If there is a point (*x*_0_^(1)^ or *x*_0_^(2)^) in [Ex_2_ − 3En_2_, Ex_1_ + 3En_1_], as shown in Figure 1(a), then,

(3) S=S1+S2=∫−∞z2ϕ(x)dx+∫z2∞ϕ(x)dx=∫−∞z2ϕ(x)dx+(1−∫−∞z1ϕ(x)dx),

where *z*_1_ = (*x*_0_ − Ex_1_)/En_1_,  *z*_2_ = (*x*_0_ − Ex_2_)/En_2_.

(3) If *x*_0_^(1)^ and *x*_0_^(2)^ are in the interval [Ex_2_ − 3En_2_, Ex_1_ + 3En_1_] simultaneously, as shown in Figures 1(b) and 1(c), then,

(4) S=S1+S2+S3={∫−∞z1(1)ϕ(x)dx+(∫−∞z2(2)ϕ(x)dx−∫−∞z2(1)ϕ(x)dx)+∫z1(2)∞ϕ(x)dxEx1>Ex2∫−∞z2(1)ϕ(x)dx+(∫−∞z1(2)ϕ(x)dx−∫−∞z1(1)ϕ(x)dx)+∫z2(2)∞ϕ(x)dxEx1≤Ex2,

where *x*_0_^(1)^ ≤ *x*_0_^(2)^, *z*_*i*_^(*j*)^ = (*x*_0_^(*j*)^ − Ex_*i*_)/En_*i*_.

Considering the normalization of the similarity, *S* must be normalized. The area is seen as the similarity of two normal distributions after it is normalized.

**Definition 1 (similarity of normal cloud)**

Given normal cloud ${\vec{C}}_{i}=\left\{{\text{Ex}}_{i},{\text{En}}_{i},{\text{He}}_{i}\right\}$, ${\vec{C}}_{j}=\left\{{\text{Ex}}_{j},{\text{En}}_{j},{\text{He}}_{j}\right\}$, their similarity degree is

(5) $$\begin{array}{c} \gamma \left({\vec{C}}_{i},{\vec{C}}_{j}\right)=\frac{2S}{\left(\sqrt{2\pi }\left({\text{En}}_{i}+{\text{En}}_{j}\right)\right)}, \end{array}$$

where *S* is the intersection area between ${\vec{C}}_{i}$ and ${\vec{C}}_{j}$.

## 3. Cloud Virtual Pan-Concept-Tree and Concept Jumping Up

### 3.1. Cloud Virtual Pan-Concept-Tree

The core idea of data mining is to discover and obtain the potential knowledge. In this process, the knowledge layers will be different with different knowledge granularity. To deal with the uncertainty of qualitative concepts, cloud model can be used to text concept representation. Text concepts set *C* is composed of a series of basic concepts, which can be represented as cloud vector *C*_*i*_(Ex_*i*_, En_*i*_, He_*i*_). On this basis, text virtual concept tree can be structured layer by layer.

The virtual concept tree, which is based on cloud model, is uncertainty. On the same layer, the distinction between the various concepts is flexible. A certain degree of overlap is allowed. In other words, the attributes with the same value may belong to different concepts and different attribute has different contribution to concepts. In the construction process, the layer of concept extraction is uncertain. It can be extracted from the bottom layer or the upper layers.

In the data mining process, with the increasing of the granularity and the abstraction degree to concept, there is no physical structure of pan-concept-tree. In the same time, there is no the process of the concept climbing and jumping up layer by layer. The granularity of concepts is continuous and can jump up to any greater granularity.

**Figure 2:**
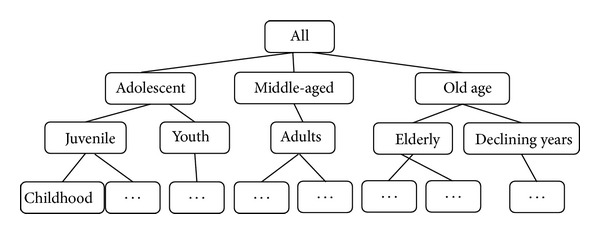
Cloud pan-concept-tree for person age distribution.

Figure 2 shows a pan-concept-tree for person age distribution based on cloud model.

**Figure 3:**
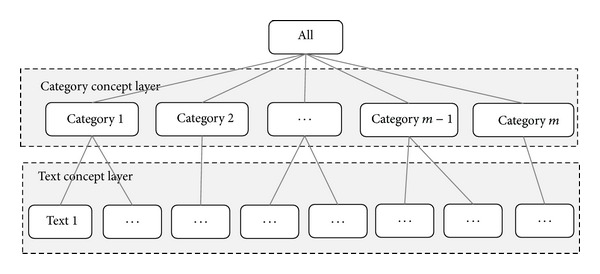
Pan-concept-tree for text classification.

On the basis of cloud pan-concept-tree, a similar pan-concept-tree model for text classification is proposed (Figure 3).

### 3.2. Concept Jumping Up and Merger

The jumping up of concept refers to directly rasie the concept to the required granularity or layer with the leaf nodes in pan-concept-tree.

The strategies for concept jumping up are as follows:

- with the user-specified amount of concept;
- automatic jumping up without the amount of concept;
- man-machine interactive jumping up.

The concept jumping up process of pan-concept-tree usually only visits the original dataset a few times. When the original dataset is large, it can reduce the computing overhead. The strategy (a) and (b) access dataset only one time and strategy (c) requires more, whose I/O overhead depends on the number of human-machine interaction. In fact, strategy (c) can be achieved by invoking strategy (a) repeatedly.

In text classification practice, strategy (a) is mainly used to concept jumping up because the number of text categories is known. In the concept jumping up process, the merger between concepts is the most important operation. It can merge two adjacent concepts and forms an upper layer (or thicker granularity) concept. In cloud model, the concept merger operations are described as follows [7, 8].

Given two adjacent concepts, which can be signed as cloud vector *C*_1_(Ex_1_, En_1_, He_1_) and *C*_2_(Ex_2_, En_2_, He_2_), the merged cloud vector is *C*(Ex, En, He), so

(6) $$\begin{array}{c} \text{Ex}=\frac{\text{E}{\text{x}}_{1}\text{E}{\text{n}}_{1}+\text{E}{\text{x}}_{2}\text{E}{\text{n}}_{2}}{\text{E}{\text{n}}_{1}+\text{E}{\text{n}}_{2}};\\ \text{En}=\text{E}{\text{n}}_{1}+\text{E}{\text{n}}_{2};\\ \text{He}=\frac{\text{H}{\text{e}}_{1}\text{E}{\text{n}}_{1}+\text{H}{\text{e}}_{2}\text{E}{\text{n}}_{2}}{\text{E}{\text{n}}_{1}+\text{E}{\text{n}}_{2}}. \end{array}$$

## 4. Text Classification Approach Based on Cloud Concept Jumping Up

In order to apply cloud model theory to text mining, we need to implement the corresponding preoperation to text, which involves the construction of text information table and conversion.

### 4.1. Text Information Table and Conversion

**Definition 2**

An information table [5] is defined as *S* = 〈*U*, *R*, *V*, *f*〉, where *U* is a nonempty finite set of objects, and *R* is a nonempty finite set of attributes, *R* = *C* ∪ *D*, where *C* is the set of condition attributes and *D* is the set of decision attributes, *D* ≠ *ϕ*. *V* = ∪*V*_*p*_, *p* ∈ *R*, and *V*_*p*_ are the domain of the attribute *p* · *f* : *U* × *R* → *V* is a total function such that *f*(*x*_*i*_, *p*) ∈ *V*_*p*_ for every *p* ∈ *R*, *x*_*i*_ ∈ *U*.

On the basis of information table, text information table is given.

**Definition 3**

Given information table *S* = 〈*U*, *R*, *V*, *f*〉, where *U* is text set, *R* = *C* ∪ *D* is a nonempty finite set of attributes, where *C* is the set of text feature attributes and *D* is the set of categories. *V* = ∪*V*_*p*_, *p* ∈ *R*, and *V*_*p*_ are the domain of the attribute *p* · *f* : *U* × *R* → *V* is a total function such that *f*(*x*_*i*_, *p*) ∈ *V*_*p*_ for every *p* ∈ *R*, *x*_*i*_ ∈ *U*.

Using cloud model to describe the uncertainty between texts, it must be met that the values of different attribute in the text belong to the same domain. That is, the values of different attribute should have the same physical meaning. But the values and the meaning of attributes of the existing text information table are so different that we must find one way to convert them to the same physical domain. In this paper, a text information table conversion algorithm is proposed using statistical method.

**Algorithm 4**

The conversion algorithm for text information table.

*Input.* text information table *S*

*Output.* conversion text information table *S*_*c*_

 Step 1. Set *S*_*c*_ = *S*
 Step 2. For each *D*_*k*_ in *D*,  *Do*//*D* is categories set of *S*_*c*_
  1. *S*_*jk*_ = ∑_*i*=1_^*m*^ *U*_*ij*_, ${\overset{-}{D}}_{jk}=\left(1/m\right)S_{jk}//m$ is the number of the texts whose category is *D*_*k*_, *U*_*ij*_ is the value of the *j*th attribute of the *i*th text.
  2. $U_{ij}=|U_{ij}-{\overset{-}{D}}_{jk}|$ //Compute fluctuation degree of the attribute values of all sample
  3. *U*_*ij*_ = *U*_*ij*_/*S*_*jk*_ // Normalization
  4. Next
 Step 3. Return *S*_*c*_.

After the conversion by Algorithm 4, the attributes of the text information table are changed into the same physical space. The novel table shows the fluctuation degree of the values in the different category and describes their statistical distribution.

### 4.2. Cloud Concept Jumping Up Classifier

**Figure 4:**
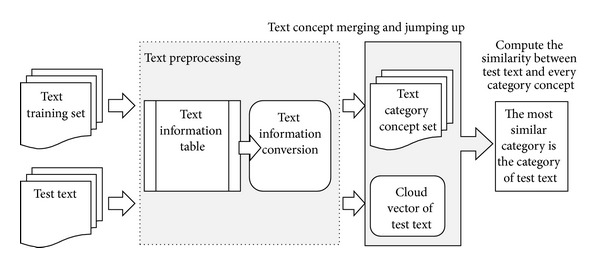
Text classification process based on cloud concept jumping up.

On the basis of text information table conversion and similarity calculation, the text classifier based on cloud concept jumping up (CCJU-TC) is proposed. The classifier works as follows (Figure 4).

Whole CCJU-TC algorithm is divided into text preprocessing, text information table conversion, category concept extraction, text cloud model conceptual similarity, and several other components.

**Algorithm 5**

CCJU-TC (The text classifier based on cloud concept jumping up)

*Input.* training text set *T*, test text *d* (unknown category)

*Output.* the category of *d*

 Step 1. Text (include *T* and *d*) segment and remove stopped items;
 Step 2. Compute the weight of items by TF-IDF [9] formula;
 Step 3. Text features (items) selection;
 Step 4. Construct information table *S* = 〈*T*, *C* ∪ *D*, *V*, *f*〉, where *C* is text feature set and *D* is category set;
 Step 5. Invoke Algorithm 4 to convert *S* to *S*_*c*_
 Step 6. Loop each category *D*_*i*_ in *D*, calculate its concept cloud vector. Finally obtain categories concept set *C*_concept_
  - For *j* = 1 to |*D*_*i*_ | −1
  - *C*_*T*_*j*__ = *C*_*T*_*j*__ MERGE  *C*_*T*_*j*+1__ //obtain category concept by concept merging, where *T*_*j*_, *T*_*j*+1_ are texts with the same category *D*_*i*_, *C*_*T*_*j*__ and *C*_*T*_*j*__ are their corresponding cloud models.
  - Next
  - *C*_concept_ = *C*_concept_ ∪ *C*_*T*_*j*__;
 Step 7. Compute max⁡(*μ*(*C*_*d*_, *C*_concet*p*_′)), where *C*_*d*_ is cloud vector of *d*, *μ*(*C*_*d*_, *C*_concet*p*_′) is the cloud similarity between *C*_*d*_ and any category concept in *C*_concet*p*_. The category with most great similarity is the category of text *d*.
 Step 8. Return.

## 5. Experiments and Evaluations

### 5.1. Experiment on Different Datasets

In order to evaluate the performance of CCJU-TC facility, we have acquired four datasets of varying characteristics. Each dataset has its own unique characteristics in terms of the degree of similarity between categories and the dimensionality of categories, as shown in Table 1.

The Featured Articles dataset was designed and organized by our research group by extracting different types of articles from Wikipedia website. A total of 1159 articles were acquired from twenty-three randomly selected categories. 10 documents from each category have been randomly selected to build the training set. The remaining documents were utilized for testing purposes. In other word, the training set of this dataset consists of 230 documents, while the testing set consists of a total of 929 documents.

The Vehicles dataset was built by extracting vehicle related articles from Wikipedia website. This dataset was acquired by extracting articles from four subcategories in the category of ‘‘Vehicles.” All the four categories are easily differentiated and each category has its own unique keywords. This dataset consists of 640 documents. Each category consists of 160 documents where 50 documents were used to build the training set and the remaining 110 documents were utilized for testing purposes. In other words, the training set consists of a total of 200 documents, while the testing set contains a total of 440 documents.

A dataset containing articles about mathematical topics has been acquired from arxiv.org. This dataset consists of eight categories regarding mathematical topics. 40 documents for each category have been collected, and the entire dataset consists of a total of 320 documents. 10 documents from each category were extracted randomly to build the training set, while the remaining 30 documents from each category were used for testing purposes.

20-Newsgroups dataset is one of the standard benchmark datasets used by many text classification research groups to evaluate the performance of their presented classification approaches. 20-Newsgroups dataset is a collection of 20,000 Usenet articles from twenty different newsgroups with 1000 articles per newsgroup. 20-Newsgroups collection has become a widely used dataset for experiments in text applications of machine learning techniques, such as text classification and text clustering. 20-Newsgroups dataset used in our experiments was acquired from the CMU Text Learning Group's website. In our experiments using this dataset, every category was divided into two subsets. 300 documents from each category were divided for training, while the remaining 700 documents were used for testing purposes. In other words, the training set consists of 6000 documents and the remaining 14,000 documents are used for testing purpose.

Many research works in text document classification apply preprocesses such as stop word elimination, word stemming, and feature selection to the datasets used in their experiments and evaluations in order to obtain better experimental results.

As our research goal in this paper is to evaluate the performance of CCJU-TC facility without sacrificing the simplicity and low cost classification algorithms, we did not perform any preprocess such as stop word elimination, word stemming to the datasets that we used in our experiments. In order to reduce the interference of different feature set, IG, CHI [21, 22], and FAS [23, 24] feature selection methods are adopted.

In all experiments, we employ SVM (Torch), *k*NN (*k* = 30), and NB classifiers as a comparison. In the way to construct the training set and test set, we apply the 3-fold cross validation, which randomly divides the text sets into three parts. Two parts are used as training sets; the other left is test set. Finally, the average of three classification results is the experimental result, which is evaluated by precision rate (*p*), recall rate (*r*), and *F*_1_(2 × *p* × *r*/(*p* + *r*)). The details of experimental result are in Tables 2, 3, and 4.

**Figure 5:**
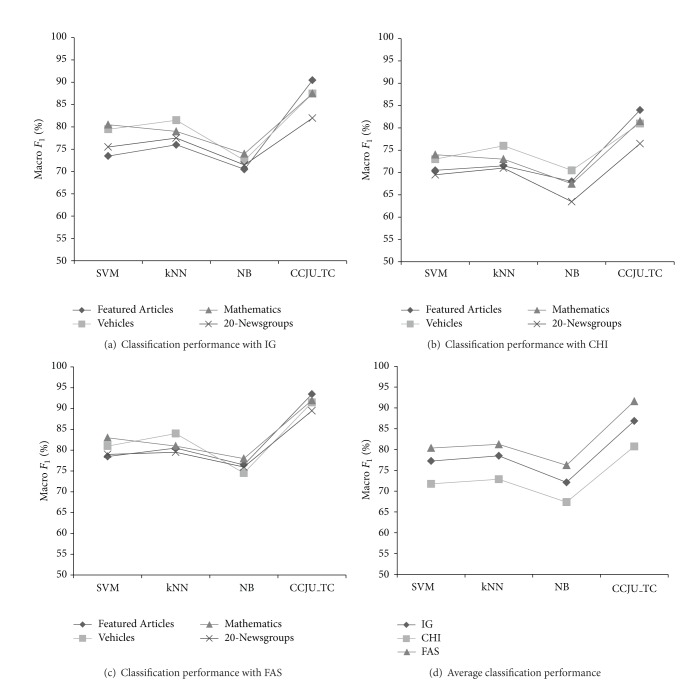
Classification results comparison.

In order to compare the performance of the classifier more intuitive, Figure 5 is created based on the above tables.

### 5.2. Summary of Experiment

In the above classification experiment, CCJU-TC has the better performance, followed by *k*NN and SVM, NB classification performance is the worst (Figure 5). Meanwhile, the different feature selection method for text categorization performance impact is also different. The performance of the text classifier, which selected features by FAS, is higher and stable (Figure 5(c)). CHI feature selection method for text classification more significant impact, mainly due to over-reliance on low-frequency features, especially in the 20-Newsgroups dataset (Figure 5(b)).

Through the comparison test among multiple text classifiers with a variety of feature selection methods on the different datasets, CCJU-TC has shown an excellent text classification capability. It not only has good ability to adapt different feature set, but also has better classification ability. It full proves that CCJU-TC is an efficient classifier.

## 6. Conclusion

In the present research to text mining (TM), traditional data mining methods still dominated. However, with further research, it faces more severe challenges. These difficulties, such as the huge dimensions and sparsity of text object, the high complexity of algorithm, and the requirement of prior knowledge, have seriously hampered the development of TM.

Through in-depth analysis, these problems in TM process are due to the uncertainty of natural language. The uncertainty of natural language (especially text) comes from the uncertainty of the human thinking in essence. Although it strengthens understanding of spatial and cognitive for people, brings a series of problems to TM. Therefore, from the point of reducing the complexity of natural language, if we can carry out the advanced innovation, which is based on making full use of these existing technologies, and find out a novel uncertainty artificial intelligence approach for TM, it will greatly facilitate the rapid development of TM.

CCJU-TC classifier is a novel attempt to apply uncertain knowledge acquisition tool (cloud model) to text classification in TM. The experimental result shows that it is an excellent solution. Future, more data mining methods, based on cloud model, will be carried out.

## Figures and Tables

**Table 1:** Datasets and their characteristics.

Datasets	Degree of similarity between categories	Number of articles	Dimensionality of categories
Featured Articles (Wikipedia)	Normal	1159	23
Vehicles (Wikipedia)	Low	640	4
Mathematics (Arxiv.org)	High	320	8
20-Newsgroups (CMU Text Learning Group)	Normal	20,000	20

**Table 2:** Classification performance of different classifier with IG.

Datasets	SVM			*k*NN			NB			CCJU-TC		
	*p* (%)	*r* (%)	*F*_1_	*p* (%)	*r* (%)	*F*_1_	*p* (%)	*r* (%)	*F*_1_	*p* (%)	*r* (%)	*F*_1_
Featured Articles	72	75	73.47	75	77	75.99	70	71	70.50	89	92	**90.48**
Vehicles	78	81	79.47	81	82	81.50	72	73	72.50	86	89	**87.47**
Mathematics	80	81	80.50	78	80	78.99	73	75	73.99	87	88	**87.50**
20-Newsgroups	75	76	75.50	77	78	77.50	70	73	71.47	81	83	**81.99**

Average	76.25	78.25	77.23	77.75	79.25	78.49	71.25	73	72.11	85.75	88	**86.86**

**Table 3:** Classification performance of different classifier with CHI.

Datasets	SVM			*k*NN			NB			CCJU-TC		
	*p* (%)	*r* (%)	*F*_1_	*p* (%)	*r* (%)	*F*_1_	*p* (%)	*r* (%)	*F*_1_	*p* (%)	*r* (%)	*F*_1_
Featured Articles	70	71	70.50	71	72	71.50	68	68	68.00	83	85	**83.99 **
Vehicles	72	74	72.99	75	77	75.99	70	71	70.50	80	82	**80.99 **
Mathematics	74	74	74.00	73	73	73.00	66	69	67.47	81	82	**81.50 **
20-Newsgroups	69	70	69.50	70	72	70.99	62	65	63.46	75	78	**76.47 **

Average	71.25	72.25	71.75	72.25	73.5	72.87	66.5	68.25	67.36	79.75	81.75	**80.74 **

**Table 4:** Classification performance of different classifier with FAS.

Datasets	SVM			*k*NN			NB			CCJU-TC		
	*p* (%)	*r* (%)	*F*_1_	*p* (%)	*r* (%)	*F*_1_	*p* (%)	*r* (%)	*F*_1_	*p* (%)	*r* (%)	*F*_1_
Featured Articles	78	79	78.50	80	81	80.50	75	78	76.47	92	95	**93.48 **
Vehicles	80	82	80.99	83	85	83.99	74	75	74.50	90	93	**91.48 **
Mathematics	82	84	82.99	80	82	80.99	77	79	77.99	91	93	**91.99 **
20-Newsgroups	79	79	79.00	79	80	79.50	74	78	75.95	88	91	**89.47 **

Average	79.75	81	80.37	80.5	82	81.24	75	77.5	76.23	90.25	93	**91.60 **
